# Enhanced Diffusion and Non-Gaussian Displacements of Colloids in Quasi-2D Suspensions of Motile Bacteria

**DOI:** 10.3390/ma17205013

**Published:** 2024-10-14

**Authors:** Xiao Chen, Yaner Yan

**Affiliations:** 1 School of Life Science, Huaiyin Normal University, Huai’an 223300, China; 2 Jiangsu Key Laboratory for Eco-Agricultural Biotechnology around Hongze Lake/Collaborative Innovation Center of Regional Modern Agriculture & Environmental Protection, Huaiyin Normal University, Huai’an 223300, China

**Keywords:** colloid, enhanced diffusion, non-Gaussian, bacteria, active matter

## Abstract

In the real world, active agents interact with surrounding passive objects, thus introducing additional degrees of complexity. The relative contributions of far-field hydrodynamic and near-field contact interactions to the anomalous diffusion of passive particles in suspensions of active swimmers remain a subject of ongoing debate. We constructed a quasi-two-dimensional microswimmer–colloid mixed system by taking advantage of *Serratia marcescens*’ tendency to become trapped at the air–water interface to investigate the origins of the enhanced diffusion and non-Gaussianity of the displacement distributions of passive colloidal tracers. Our findings reveal that the diffusion behavior of colloidal particles exhibits a strong dependence on bacterial density. At moderate densities, the collective dynamics of bacteria dominate the diffusion of tracer particles. In dilute bacterial suspensions, although there are multiple dynamic types present, near-field contact interactions such as collisions play a major role in the enhancement of colloidal transport and the emergence of non-Gaussian displacement distributions characterized by heavy exponential tails in short times. Despite the distinct types of microorganisms and their diverse self-propulsion mechanisms, a generality in the diffusion behavior of passive colloids and their underlying dynamics is observed.

## 1. Introduction

Active matter systems, composed of self-propelled agents, are prevalent across a wide range of natural phenomena, from the microscale to the macroscale. These systems include cytoskeletal filaments [1], bacteria [2,3], algae [4], sperm cells [5], fish schools [6], and bird flocks [7]. The agents within these active systems generate internal driving forces exerted on specific degrees of freedom and continuously exchange energy with their environments, which propels the system far from equilibrium, exempting it from conventional equilibrium constraints such as the detailed balance condition [8] and the fluctuation–dissipation theorem [9]. As a result, active matter exhibits a broader and more intricate array of physical behaviors than those typically observed in thermodynamic systems at equilibrium.

In nature, nearly all swimming microorganisms inevitably interact with dispersed particles in their surroundings, which from a physics perspective can be viewed as the interplay between a passive colloid and self-propelled units comprising an active material. The microswimmers act like a thermal bath, leading to universal two-timescale dynamics where the colloid exhibits ballistic motion over short times, transitioning to diffusive behavior over longer times. However, the diffusion of passive particles in suspensions of active agents deviates significantly from classical Brownian motion driven by thermal fluctuations, as evidenced by the enhancement in diffusivity [10,11,12,13,14,15], which can be up to 1–3 orders of magnitude higher than the Brownian diffusion coefficient [16,17,18,19], and the anomalous non-Gaussian displacements, exhibiting heavy exponential [10,17,18,20] or power [14,19,21] tailed distributions. On short timescales, non-Gaussian and superdiffusive behavior is often observed, but, on long timescales, repeated uncorrelated interactions between active matter and passive units lead to enhanced diffusion dynamics characterized by Gaussian displacements.

The pioneering experiments by Wu and Libchaber were the first to demonstrate that colloidal particles in bacterial suspensions exhibit persistent random walks with diffusivity up to even hundreds times higher than that predicted by classical Brownian motion [16], laying the foundation for studies on active transport. Although the diffusion of particles over long times is similar to Brownian motion, the physical origin of the large positional fluctuations is different and attributed to the collective dynamics of bacteria. In suspensions of the swimming alga *Chlamydomonas reinhardtii*, which are much more dilute than the bacterial solutions studied by Wu [16], Leptos et al. observed that the tracer trajectories involve both Brownian components and large displacements [10]. They attributed the observed enhanced diffusion to the far-field advection induced by individual swimmers [10], a hypothesis on hydrodynamic interaction subsequently corroborated by further experimental findings in dilute microswimmer suspensions [13,21]. However, recent experiments by Jeanneret et al. have shown that the dominant mechanism underlying the enhancement of colloidal diffusion is a jump-diffusion process, which arises from the entrainment of colloidal particles by swimming algae, necessitating head-on collisions [18]. Other experiments and theoretical models based on purely steric interactions also suggest that the origin of enhanced diffusion is largely dependent on near-field contact interactions, such as collision processes [17,22]. Despite significant advances in understanding the dynamics of colloids in active particle suspensions, critical aspects of this field remain elusive and contentious.

Here, we introduce a quasi-two-dimensional experimental system close to the air–water interface with variable densities of swimmers, composed of a mixture of motile bacteria and tracer colloids, to investigate the anomalous diffusion behavior of colloidal particles and its underlying mechanisms. This study also has broader implications for understanding biological and ecological processes, including the nutrient transport and uptake by microorganisms accumulating near surfaces [15,23,24] and the diffusion of floating microplastics in the marine environment [25], and medical applications, such as developing drug-delivery micromachines [26,27].

## 2. Materials and Methods

### 2.1. Experimental Setup

*Serratia marcescens* (ATCC 274) is a rod-shaped, flagellated bacterium. A small amount bacteria of the frozen stock was first inoculated into 4 mL of Terrific Broth (Sangon Biotech). This initial culture was incubated overnight at 30 °C. Then, 500 μL of the overnight culture (optical density OD650≈1.0) was transferred into 10 mL of fresh Terrific Broth and incubated at 33 °C with shaking at 200 rpm for 2.5 h. The cultured bacterial suspension was then diluted 1:1 with deionized water, resulting in a final bacterial concentration of approximately $10^{7}$ cells/mL in solutions. At this stage, the aspect ratio of cell bodies was around 3.

**Figure 1:**
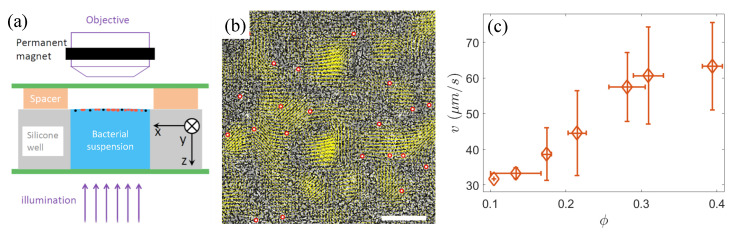
(**a**) The schematic of the experimental setup. The red and black solid circles near the surface of bacterial suspension represent the bacteria and passive tracers, respectively. (**b**) Instantaneous velocity field of bacteria (yellow arrows) at an area fraction of ϕ≈0.4, overlaid on the raw experimental image. The dark rod-shaped clusters in the raw image are *S. marcescens* bacteria. The red circles indicate the identified tracer colloids. Scale bar = 50 μm. (**c**) The mean speed *v* of bacteria as a function of the area fraction ϕ.

In the diluted bacterial solution, a small amount of 2.8 μm diameter superparamagnetic beads (Invitrogen Dynabeads) was added as tracer particles. The mixture suspension containing the tracers was placed into a closed chamber constructed from a glass slide, cover slip, and a silicone spacer (Figure 1a). The spacer had an inner diameter of 15 mm and a depth of 2 mm. Due to the hydrophobic nature of the bacterial surface [28,29], *S. marcescens* cells tended to migrate to the air–liquid interface and adhere to the water surface, gradually forming a monolayer bacterial film with increasing density, which enabled the establishment of a bacterial density gradient. We also found that, if a small amount of the surfactant Brij-35 is added to the bacterial suspension, *S. marcescens* can easily escape from the confinement and return freely to the bulk liquid. However, *S. marcescens* needs to have its flagella bundled in a water environment to propel itself at normal swimming speeds. Therefore, we believe that the majority of the bacterial bodies remain submerged below the water surface.

As superparamagnetic beads exhibit magnetic behavior only in the presence of an external magnetic field, a vertically movable ring magnet was fixed above the stage of an upright microscope (Nikon ECLIPSE Ni) to attract the beads to the interface. The ring magnet was positioned around the objective lens (Figure 1a), aligning the central axis of the ring magnet with the optical path of the lens, ensuring that the observation region of the sample was directly below the center of the magnet, where the magnetic field lines are oriented nearly vertically (i.e., parallel to the z-axis). When the magnetic field is removed, the majority of the beads fall from the liquid surface. Thus, we consider that most beads are very close to the interface but still below the water surface. As a result, a quasi-two-dimensional system consisting of a mixture of active microswimmers and passive colloids was constructed. Furthermore, the bead concentration was kept very low, with an average distance of approximately 50 μm between neighboring beads to minimize their interactions. Additionally, the observed central region of the sample (960 × 480 μ $m^{2}$ under a 20× phase-contrast objective lens) was sufficiently small compared to the area of the central opening of the ring magnet, allowing us to assume that the observation area was flat and the horizontal component of the magnetic field was negligible. These all avoid the chain aggregation of the magnetized beads near the interface (Figure 1b). The image data at different bacterial densities were acquired using a Basler camera (Basler acA2040-180 km, Ahrensburg, Germany) at a frame rate of 90 frames/s. Each video was recorded for 25 s, during which the bacterial density at the air–liquid interface remained relatively stable, indicating a quasi-steady state.

### 2.2. Image Analysis

We utilized the image processing technique outlined in Ref. [30] to isolate and track bacterial motion. Initially, a moving Hamming window was applied to smooth the raw image, generating a background image, which was then subtracted from the original image. Bacterial edges were then identified using a gradient-based edge detection algorithm, with the pixels inside the detected edges set to white, defining the area fraction, ϕ, as a measure of bacterial density. To enhance the bacterial edges and eliminate lighting inconsistencies, a high-pass filter was applied, followed by a median filter to remove noise. The denoised grayscale image underwent morphological opening, closing reconstruction, and multiple erosion operations to isolate individual bacteria. The bacterial properties, including centroid position and size, were then identified using Matlab’s regionprops function. These properties were tracked with a custom particle tracking algorithm based on the method described in [30], enabling the calculation of the instantaneous velocity field of bacteria in dense populations.

For tracking the sparse tracer beads, we employed the Particle Tracking software (https://physics.emory.edu/faculty/weeks/idl/) developed by Crocker and Grier [31], adjusting parameters appropriately. Figure 1b shows the instantaneous velocity field of the bacteria and the identification of tracer colloidal particles at a bacterial area fraction of ϕ≈0.4.

## 3. Results

Given that bacteria are posited as an active thermal bath driving colloidal diffusion, we first measured the mean speed *v* of bacteria at area fractions ϕ ranging from 0.1 to 0.4. The sparse bacterial suspension (ϕ≈0.1) can be likened to a dilute gas composed of weakly interacting motile bacteria, with an average speed of approximately 30 μm/s. As the bacterial density increases, intensified local interactions lead to enhanced collective behavior, causing the emergence of jet- or vortex-like structures, analogous to those observed in classical hydrodynamic turbulence (see Figure 1b). Within these localized structures, bacterial motion tends to become highly aligned, often reaching velocities significantly surpassing those of individual free-swimming bacteria, resulting in a concomitant increase in the average speed *v* (Figure 1c).

**Figure 2:**
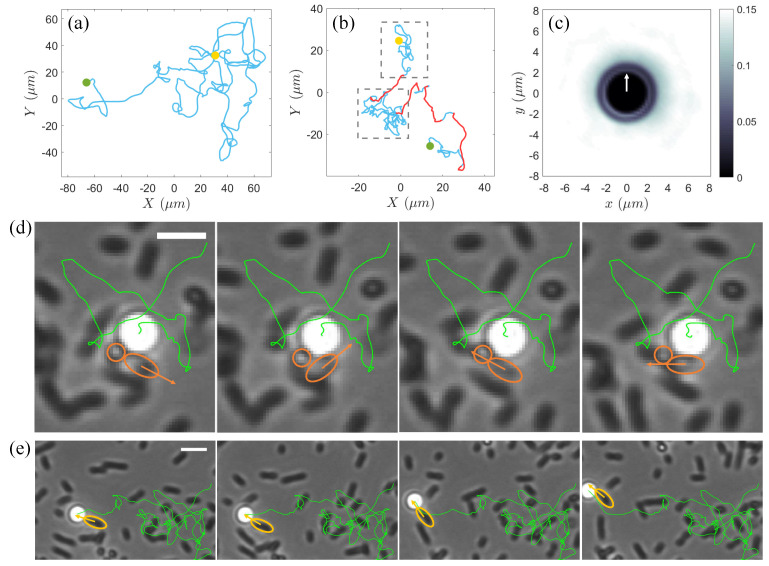
(**a**) A typical trajectory (blue solid line) of a colloidal particle over 25 s at an area fraction ϕ≈0.4. (**b**) Trajectory of a colloidal particle over 25 s in a bacterial suspension at an area fraction ϕ≈0.1. The gray dashed rectangle highlights a region with localized random tracks (blue solid line), while the red solid lines indicate the large jumps. In (**a**,**b**), the yellow and green solid circles mark the starting and end points of the trajectories, respectively. (**c**) Two-dimensional spatial distribution of bacteria around colloids in the moving coordinate frame of colloid during large jumps. The white arrow indicates the instantaneous motion direction of the colloid. (**d**) Time series over a total duration of 0.07 s of a small semi-loopy trajectory of a colloidal particle influenced by two self-spinning cells (encircled by orange solid lines) in a dilute bacterial suspension of ϕ≈0.1. The body orientation of the short-rod-shaped one of the two spinning bacteria is marked by an orange arrow. The green solid line displays the entire trajectory of the colloid from entering the field of view until the end of this semi-loopy track event. Scale bar = 4 μm. (**e**) Time series over a total duration of 0.2 s of a large jump in the trajectory (green solid line) of the same colloid in (**d**) propelled by a bacterium (encircled by yellow solid line). The motion direction of the bacterium is indicated by a yellow arrow. Scale bar = 5 μm.

Driven by active turbulence, the tracks of tracer colloids over sufficiently long times in a moderately dense bacterial suspension consist of long, straight segments of varying lengths in random directions, resembling Brownian behavior (Figure 2a). In contrast, in dilute suspension of motile bacteria, the tracer particles display a broader spectrum of motion types due to a combination of dynamic effects (Figure 2b). The weakest of these is thermal noise caused by Brownian motion, which, even at low bacterial densities, is often imperceptible owing to the frequent disturbances from nearby swimmers. When bacteria approach the colloids without direct contact, their far-field hydrodynamic flows induce loop-like trajectories, as documented in previous studies [10,13,18,21]. However, additional sources of looped trajectories were revealed in our experiments. Figure 2d illustrates the influence of the rotating bacterial cells on the tracks of nearby particles within the near field. In contrast to the rod-shaped bacteria, spherical-like *S. marcescens* trapped at the air–water interface exhibit counterclockwise self-spinning about the vertical (z-) axis owing to the bundling of their flagella perpendicular to the horizontal plane [32]. In this experimental system, due to the non-uniformity in the aspect ratios of rod-shaped cell bodies, a small number of spherical-like or short-rod-shaped bacteria also display rotating behavior. The rotating cell generates a horizontal rotational flow field and hydrodynamic attraction near the interface [32]. Such an effect induced by cell spinning can lead to erratic small loopy or semi-loopy trajectories in place, as shown in Figure 2d. More commonly, colloidal particles within the near field are continuously struck by surrounding bacteria swimming in random directions, causing the particles to be ejected in various directions over small time intervals. These combined mechanisms give rise to localized, chaotic random walks indicated by the blue trajectories highlighted within the gray dashed rectangle in Figure 2b.

On the other hand, when a particle is subjected to collisions and sustained pushing by one or several bacteria moving in a single direction, it can undergo a jump-like large displacement (Figure 2e and the red solid line in Figure 2b). We calculated the two-dimensional spatial distribution of the bacteria around the colloid during these jumps and found that, within an approximately 3 μm range from the colloid surface, the spatial distribution of the bacteria is anisotropic, with a higher probability of being found in the direction opposite to the instantaneous velocity of the colloid (Figure 2c). This supports that the long-range migration is predominantly driven by near-field collisions and entrainments by bacteria.

**Figure 3:**
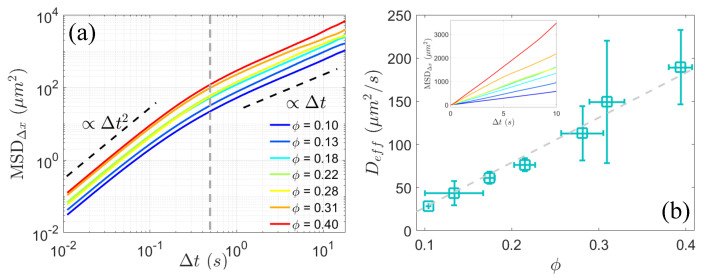
(**a**) Mean squared displacement (MSD) of colloidal particles along x-axis in the laboratory frame for various bacterial area fractions ϕ. After a lag time of Δt≳0.5 s (indicated by the gray dashed line), the scaling of MSD transitions from 2 to 1. (**b**) Effective diffusion coefficient $D_{\mathrm{eff}}$ (square □), obtained by fitting the MSD in linear coordinates shown in the inset with ${\mathrm{MSD}}_{\Delta x}=2D_{\mathrm{eff}}\Delta t$ as a function of area fraction ϕ. The gray dashed line represents a linear fit to the data.

To investigate the anomalous diffusion dynamics of passive colloids, we computed the mean squared displacements (MSDs) along the x-axis in the laboratory frame, denoted as ${\mathrm{MSD}}_{\Delta x}=\langle \Delta x{\left(\Delta t\right)}^{2}\rangle$, where Δt is the lag time, Δx represents the x-component of displacements of colloids, and 〈⋯〉 indicates the ensemble average. ${\mathrm{MSD}}_{\Delta x}$ exhibits the general two-timescale dynamic regimes as shown in Figure 3a. On short timescales (Δt<0.2 s), the system displays superdiffusive behavior with a scaling exponent of 2 across all density conditions. This suggests that colloidal transport is ballistic in nature over small time intervals, primarily due to near-field contact interactions between swimmers and passive particles. However, under multiple uncorrelated interactions between bacteria and colloidal particles over a large lag time more than about 0.5 s, MSD transitions to a scaling exponent of 1 (Figure 3a), indicative of Brownian-like diffusion behavior. Based on the linearity observed in long-time diffusion (Figure 3a and the inset in Figure 3b), an effective diffusion coefficient, $D_{\mathrm{eff}}$, can be defined as $D_{\mathrm{eff}}=\langle {\left(\Delta x\right)}^{2}\rangle /2\Delta t$. As shown in Figure 3b, $D_{\mathrm{eff}}$ increases linearly with area fraction ϕ of bacteria, in agreement with previous findings [10,16,17,18,21,22], and is significantly larger than the thermal diffusivity, which is measured to be approximately 0.15 μm²/s at room temperature, indicating anomalously enhanced diffusion. With the onset of pronounced active turbulence as area fraction ϕ exceeding 0.25, $D_{\mathrm{eff}}$ rises above 100 μm²/s, consistent with the results reported by Wu in bacterial suspensions exhibiting collective dynamics [16].

**Figure 4:**
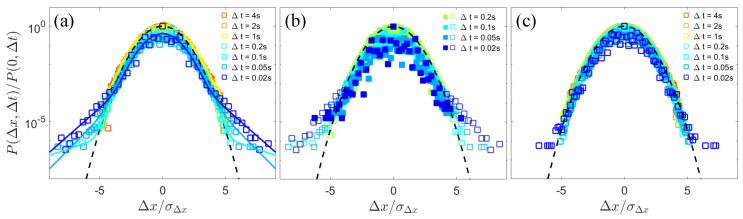
The normalized probability density function (PDF) of the x-component displacements of colloidal particles in the laboratory frame over different lag times Δt (open squares). $\sigma_{\Delta x}$ is the standard deviation of the x-component displacements. (**a**) The displacement PDF at a bacterial area fraction ϕ≈0.12. The solid lines represent the fits to the short-time displacement distributions using Equation (1) in the main text. (**b**) The short-time displacement PDF (open squares) and the PDF after removing large jumps (solid squares) at a bacterial area fraction ϕ≈0.12. (**c**) The displacement PDF at a bacterial area fraction ϕ≈0.4. The black dashed lines in (**a**–**c**) represent Gaussian fits.

We further analyzed the probability density function (PDF) of the x-component displacements of colloidal particles in the laboratory frame at different time intervals, Δt, which provides additional insights into the dynamics. As shown in Figure 4a, for the dilute bacterial suspension, the PDF of the displacement over small lag times can be described by a Gaussian center with exponential tails [10],

(1) $$P\left(\Delta x,\Delta t\right)=\frac{1-a}{\sqrt{2\pi {\delta_{g}}^{2}}}e^{-{\left(\Delta x\right)}^{2}/2{\delta_{g}}^{2}}+\frac{a}{2\delta_{e}}e^{-\left|\Delta x\right|/\delta_{e}},$$

where the standard deviation $\delta_{g}$ of the Gaussian diffusion, the characteristic length $\delta_{e}$ of the enhanced displacements, and the fractional contribution *a* of the non-Gaussian component owing to enhanced transport are fitting parameters. If removing the large jumps observed in Figure 2b, the PDF for short-time displacements tends towards a Gaussian core (Figure 4b), confirming that the non-Gaussian nature of the diffusion behavior originates from near-field collisions and the entrainments of colloids by microswimmers. With the time interval Δt increasing to over 0.2 s, the PDF gradually converges to a Gaussian distribution due to the uncorrelation of multiple interactions regarding long times. As area fraction ϕ of bacteria increases up to 0.4, the non-Gaussianity of displacements significantly diminishes (Figure 4c), consistent with our observation of Brownian-like trajectories of the passive colloids induced by active turbulence.

## 4. Discussion and Conclusions

Various studies have shown that the diffusion behavior of colloids in active suspensions is influenced by factors such as the type of swimming particles [33,34], the concentration of microswimmers, and the size ratio between the microswimmers and passive objects [9,11,12,13,17,21,35,36,37]. Here, we investigated the dynamics and statistical characteristics of the diffusion of the dispersed colloidal particles near an air–water interface, which are comparable in size to the swimming microorganisms. We found that, as bacteria transition from a sparse state to collective motion with the area fraction ϕ of the bacteria increasing from 0.1 to 0.4, the diffusion behavior of passive colloids changes accordingly. In dilute bacterial suspensions, the trajectories of colloidal particles often exhibit localized Brownian-like random walks with loop-like perturbations, followed by rare large jumps. These jumps dominate the significant enhancement in the diffusion of colloids within dilute bacterial suspensions, causing their short-time displacement distributions to deviate from paradigmatic Gaussianity and display strong exponential tails, a characteristic observed in systems with different types of microorganisms [10,17,20,38]. Furthermore, we found that these large jumps are predominantly caused by near-field collisions and pushing by bacteria, resembling the jump-diffusion process induced by the microalgal entrainment of micro-objects [18,19], although the influence of far-field hydrodynamic interactions on colloidal trajectories cannot be entirely excluded. As a result, the effective diffusion coefficients of the colloids in our experiment and observed in Refs. [18,19] are one to two orders of magnitude higher than those in systems of comparable scale [10,11,13,21] where the tracer diffusion is primarily enhanced by far-field flow induced by swimming microorganisms. This may suggest a generality in the underlying mechanism for similar enhanced diffusion behaviors of colloidal particles in swimmer–colloid mixed systems.

To conclude, the enhanced diffusion of passive colloids originates from distinct dynamics that depend on the density of microswimmers. In suspensions with moderate bacterial densities, spontaneous collective dynamics of bacteria, known as active turbulence, drive the enhancement of tracer diffusivity. However, at low bacterial densities, the colloid particles exhibit large jumps in their trajectories, predominantly arising from near-field contact interactions, along with a non-Gaussian displacement distribution characterized by heavy exponential tails. These results provide broad insights for fundamental research as well as applications across multiple fields.

## Data Availability

The authors declare that the main raw data supporting the findings of this study are available from the corresponding author upon request.
